# Role of Phosphodiesterases in Biology and Pathology 2.0

**DOI:** 10.3390/ijms25105339

**Published:** 2024-05-14

**Authors:** Mauro Giorgi, Manuela Pellegrini, Mara Massimi

**Affiliations:** 1 Department of Biology and Biotechnology “C. Darwin”, Sapienza University of Rome, Piazzale A. Moro 5, 00185 Rome, Italy; mauro.giorgi@uniroma1.it; 2 Institute of Biochemistry and Cell Biology, IBBC-CNR, Via E. Ramarini 32, 00015 Monterotondo (RM), Italy; 3 Department of Life, Health and Environmental Sciences, University of L’Aquila, Via Vetoio, 67100 L’Aquila, Italy

Phosphodiesterases (PDEs) are ubiquitous enzymes that hydrolyse cAMP and cGMP second messengers temporally, spatially, and integratedly according to their expression and compartmentalization inside the cell. Eleven families of PDEs (PDE1-PDE11) have been described; some of them are selective for cAMP (PDE4, PDE7, PDE8) or cGMP (PDE5, PDE6, PDE9), while others function on both cyclic nucleotides [1].

PDEs may form specific multi-molecular complexes, which allow the propagation of signals along well-defined pathways and prevent their diffusion, ensuring the specificity of downstream biological responses [2]. As cAMP and cGMP signalling regulate a huge variety of cell functions, PDEs are involved in many aspects of cell physiology, and their modifications are of considerable importance in human pathology. A great number of PDE inhibitors have been developed in the past few decades and successfully used in the treatment of impotence and chronic pulmonary diseases, while new evidence underlines the possible use of these inhibitors in the treatment of cardiovascular disease, type 2 diabetes, cancer, inflammation, and other pathologies [3,4].

The present Special Issue in *IJMS,* entitled “Role of Phosphodiesterase in Biology and Pathology 2.0”, includes a total of 10 contributions (six original articles and four reviews), providing new information on different aspects of the widespread role of PDEs, of their isoforms, as well as of their specific inhibition, in different physiological and pathological conditions, such as cardiac contractility, melanoma, skeletal muscle function and structure, parasitosis, systemic and portal blood pressure, and liver cirrhosis.

A relevant pathologic aspect, which is becoming increasingly common within the ever-growing global elderly population, is the use of inhibitors of PDEs involved in the control and modulation of neurodegenerative pathologies with particular attention to the cAMP specific PDEs, such as PDE4, PDE7, and PDE8. In this Special Issue, the review by Mussen and collaborators (Contribution 1) attributed particular attention to the role of PDE inhibitors, responsible for the anti-inflammatory PDE response during spinal cord repair following traumatic spinal cord injury, and to non-selective inhibitors capable of reducing the degenerative progression of multiple sclerosis (MS), a chronic disease that presents neurological and autoimmune characteristics, making the disease difficult to treat.

Benítez-Fernández et al. (Contribution 2) investigated P3.15, a small molecule that shows a dual function of inhibiting glycogen synthase kinase 3β and PDE7. VP3.15 ameliorates the pathology course by improving motor deficits and affecting the immunomodulatory response of mice with primary–progressive MS. Moreover, it shows significant efficacy in the proliferation and differentiation of oligodendroglial precursors, improving the preservation of myelin and axonal integrity, suggesting that VP3.15 could be used for an integrative therapeutic strategy of the disease.

Another important aspect developed in this Special Issue concerns the involvement of the cGMP-specific PDE5A in the control of cellular metabolism and in the response to redox stress. PDE5A is the main enzyme responsible for the hydrolysis of cGMP, and its specific inhibition by sildenafil has proved to be a promising therapeutic approach for the treatment of metabolic disorders in many organs and tissues [5,6].

Cardarelli and co-workers (Contribution 3) using Kluyveromyces lactis yeast as a model organism, hypothesised a specific role for each isoform (A1, A2 and A3) of murine PDE5. These isoforms differ only in the N-terminal peptide, which seems to also be responsible for the tetrameric/dimeric assembly of murine isoforms. The authors showed that this peptide can direct the mature PDE5A protein into the mitochondrial (A2) or cytosolic (A1 and A3) compartments, altering both the cAMP/cGMP and NAD(P)/NAD(P)H balance, and in this way linking intracellular cyclic nucleotides ratios with energy homeostasis and the redox state. Subsequent mass spectrometry and native PAGE studies after treatment with reducing and oxidizing agents showed that a few specific cysteine residues are responsible for the PDE5A isoform oligomeric assembly, and that enzymatic activity is lost when modified [7].

Among skeletal muscle diseases, muscular dystrophy was reported to be ameliorated following PDE5A inhibition [8], and, here, De Arcangelis et al. (Contribution 4) analyzed the expression of the three murine Pde5a isoforms in the pathophysiology of skeletal muscle and found an increased expression of the Pde5a1 isoform, most likely due to the increased expression of MyoD and Runx1 genes, in pathological muscle.

It is well known that cAMP and cGMP second messengers modulate cardiac contractility in the heart through several mechanisms [9,10]. Calamera et al. (Contribution 5) in their review, widely described the impact of different phosphodiesterases, such as PDE2, PDE3, PDE4, PDE5, and others, on cardiac contractility in normal and pathological conditions and their contribution to the regulation of the cardiac compartmentalized signalling of cAMP and cGMP.

PDE5 inhibition can also lower systemic and organ blood pressure [11,12]. In this context, Lazaro et al. (Contribution 6) demonstrated a close relationship between changes in systemic arterial pressure and changes in portal venous pressure in rats with healthy livers. PDE5 inhibition, acting on the intra-sinusoidal NO-cGMP signalling system, can lower portal blood pressure with an effect that exceeds the effect on systemic blood pressure, thus deserving further attention for all the pathologies linked to portal hypertension (PHT). PHT is considered a very serious pathology, as it can be related to hepatic encephalopathy, hepato-renal, and hepato-pulmonary syndrome, as well as to most major complications found in liver cirrhosis [13,14]. This topic is also thoroughly reviewed by Kreisel et al. (Contribution 7). The authors highlighted that in a cirrhotic liver the key enzymes endothelial NO synthase, soluble guanylate cyclase, and PDE5 are overexpressed, leading to decreased levels of cGMP. They suggest that PDE5 inhibition can induce the dilation of hepatic sinusoids and, consequently, causes a decrease in intrahepatic resistance and portal pressure in cirrhotic portal hypertension, contributing positively to the reversal of liver cirrhosis and fibrosis.

In the last 40 years, various papers have shown that the increased level of intracellular cyclic nucleotides, caused by PDE inhibitors or cycling nucleotide signalling activators, is associated with blocks of cell growth in a number of neoplastic diseases, including hepatocarcinoma, neuroblastomas, and colorectal cancers [15,16,17]. In this Special Issue, Matarrese et al. (Contribution 8), using an in vitro model of melanoma (B16F10 cells), found that β2-AR stimulation, is able to block the anti-proliferative effect associated with α2-AR stimulation, highlighting a crosstalk between the two adrenergic signaling pathways and revealing the existence of a novel pharmacological intervention for this type of tumour.

Another important aspect concerns the use of PDE4 inhibitors against the etiological agents that cause parasitosis, one of the most neglected but impactful diseases globally [18]. In this Special Issue, Zheng Yang et al. (Contribution 9) tested two PDE4 inhibitors (rolipram and NPD-0001), obtaining no significant results for nematode survival and oviposition. It cannot be ignored that currently available inhibitors, although they show a high affinity for mammalian PDE4, are not able to block nematode PDE4, suggesting the need for research into new inhibitors specific for mammalian parasitic organisms.

In mammals, numerous experimental and clinical studies have shown that PDE4 regulates fundamental functions, and its inhibition has huge therapeutic potential for different pathological conditions [19,20,21]. However, due to their wide cellular and tissue distribution, significant side effects are possible and require careful evaluation. The formulation of a specific tissue and cellular delivery for the required therapeutic purposes remains a problem to be solved. In this context, the review of Schick and Schlegel (Contribution 10) focused on the most important and recent findings dealing with PDE4 inhibition viewing them as a promising but also very challenging tool for various therapeutic approaches.

The editors are grateful to all the authors for sharing their novel findings or reviews, and to all the referees for their invaluable support in processing the manuscripts.

We believe that the articles selected for this Special Issue provide novel insights into the role of cAMP and cGMP, and of their modulation with PDEs and by PDE inhibitors, while demonstrating the key underlying molecular mechanisms of PDE action in a range of physiological and pathological conditions. We hope that the results and information reported here can encourage future studies on PDE complexes and on the clinical therapeutical combinations of different PDE inhibitors with other bioactive molecules. In-depth analyses on these topics will also aim to discover new inhibitors and develop new strategies for more specific delivery, with a reduction in the potential side effects, thus broadening their application for pathologies that are currently still seeking additional treatments.
